# A Case of Salt-Wasting Congenital Adrenal Hyperplasia Caused by a Rare Intronic Variant in the *CYP21A2* Gene

**DOI:** 10.3390/ijms26146648

**Published:** 2025-07-11

**Authors:** Zoia Antysheva, Anton Esibov, Ekaterina Avsievich, Ekaterina Petriaikina, Vladimir Yudin, Anton Keskinov, Sergey Yudin, Dmitry Svetlichnyy, Julia Krupinova, Aleksey Ivashechkin, Yulia Katsaran, Mary Woroncow, Veronika Skvortsova, Viktor Bogdanov, Pavel Volchkov

**Affiliations:** 1 Federal Research Center for Innovator and Emerging Biomedical and Pharmaceutical Technologies, 125315 Moscow, Russia; 2 Moscow Center for Advanced Studies, 123592 Moscow, Russia; 3 Moscow Clinical Scientific Center n.a. A.S. Loginov, 111123 Moscow, Russia; 4 Federal State Budgetary Institution «Centre for Strategic Planning and Management of Biomedical Health Risks» of the Federal medical and biological agency (Centre for Strategic Planning of the Federal medical and biological agency), 119121 Moscow, Russia; 5 Faculty of Medicine, Lomonosov Moscow State University, 119991 Moscow, Russia; 6 The Federal Medical Biological Agency (FMBA of Russia), 123182 Moscow, Russia

**Keywords:** *CYP21A2*, congenital adrenal hyperplasia, intronic variant, splicing mutations, adrenal insufficiency, NGS, amplicon sequencing

## Abstract

This case report describes a novel intronic mutation, *CYP21A2*:c.738+75C>T (rs1463196531), identified in a 4-year-old male with congenital adrenal insufficiency, and expands the known mutation spectrum associated with this condition. The patient, born full-term to unrelated parents, presented with adrenal failure within the first month of life, characterized by acute adrenal crisis symptoms such as vomiting, dehydration, weight loss, hypotension, and electrolyte imbalances. Hormonal evaluations confirmed primary adrenocortical insufficiency, necessitating ongoing hydrocortisone and fludrocortisone therapy. Using family trio-based amplicon sequencing of the *CYP21A2* gene, we identified compound heterozygosity consisting of a full gene deletion and a novel pathogenic intronic mutation. Additionally, analysis of WGS data was performed to rule out pathogenic variants in genes that might lead to a similar phenotype, thereby eliminating the possibility of other genes contributing to the proband’s disease. This case demonstrates the potential of using amplicon sequencing in molecular genetic diagnostic testing to detect rare intronic variants in the *CYP21A2* gene in cases of early-onset adrenal failure. It also contributes to a better understanding of the genetic basis of congenital adrenal hyperplasia (CAH), which remains a significant autosomal recessive disorder affecting cortisol and aldosterone production, with an incidence of 1 in 10,000 to 1 in 15,000 globally.

## 1. Introduction

Congenital adrenal hyperplasia (CAH) refers to a collection of autosomal recessive disorders that result in reduced cortisol and aldosterone production due to disrupted steroid conversions [1]. The most prevalent type is the 21-hydroxylase deficiency, which accounts for about 95% of CAH cases [2]. This deficiency leads to a lack of cortisol and aldosterone resulting in an excess of androgens. CAH manifests in three main clinical forms: classic forms (salt-wasting and simple virilizing types) and a non-classic form known as late onset, depending on the degree of enzyme deficiency. In cases where an individual has two different alternative alleles (compound heterozygous), it is generally believed that the phenotype is mostly determined by the allele that is less severely affected. The incidence of CAH ranges from 1 in 10,000 to 1 in 15,000, with variations among different ethnic groups [3]. In the Russian Federation, the prevalence of CAH is 1:9500 according to neonatal screening data [4].

The enzyme responsible for 21-hydroxylation, known as CYP21, is a cytochrome P450 enzyme found in the endoplasmic reticulum. It plays a crucial role in converting 17-hydroxyprogesterone to 11-deoxycortisol and progesterone to deoxycorticosterone. In individuals with 21-hydroxylase deficiency, cortisol and aldosterone synthesis may vary depending on the specific mutation. This deficiency triggers an increase in corticotropin and corticotropin-releasing hormone, leading to hyperplasia of the adrenal cortex. Such alterations result in the overproduction of cortisol precursors like progesterone, 17-hydroxyprogesterone, and adrenal androgens, as well as aldosterone precursors. In the salt-wasting form, the accumulation of these precursors intensifies the consequences of aldosterone deficiency, including low blood volume and elevated renin levels. Aldosterone enhances sodium reabsorption in the kidneys and promotes potassium excretion. However, when there is a deficiency of aldosterone, the body struggles to eliminate potassium, which can cause potassium levels to rise in the bloodstream, leading to hyperkalemia [5].

Deficiency in the 21-hydroxylase enzyme is primarily linked to mutations in the *CYP21A2* gene, part of the cytochrome P450 family. This gene is located on the short arm of chromosome 6p21.3, near the *CYP21A1P* pseudogene, which shares 98% of its coding sequence with *CYP21A2* [6]. Common mutations, including large deletions, arise from unbalanced crossing over during meiosis, which can transfer segments from pseudogenes to active genes, leading to their inactivation [7]. Only about 5% of mutations arise independently of the pseudogene. Although there is often a strong relationship between genotype and phenotype, it can be challenging to correctly associate new mutations with the phenotype observed in the patient [8].

This article presents the case of a 4-year-old male patient with a whole gene deletion of one copy of the *CYP21A2* gene and an intronic mutation rs1463196531 in a hemizygous state on the remaining allele, which led to the development of salt-wasting CAH.

## 2. Detailed Case Presentation

### 2.1. Clinical Evaluation

Our patient was the first male child born to non-related parents, delivered at 39 weeks of gestation. He weighed 3310 g and measured 50 cm in length. Upon physical examination, his vital signs were normal, and there were no signs of dysmorphia. The pregnancy proceeded without complications. There was no family history of congenital adrenal hyperplasia or virilization in female relatives.

At 14 days old, the infant was admitted to the neonatal unit due to severe vomiting and increasing lethargy. Laboratory tests indicated serum sodium levels of 120 mmol/L (normal range: 132–147 mmol/L), potassium levels of 9 mmol/L (normal range: 3.6–6.1 mmol/L), and hypoglycemia of 2.5 mmol/L (normal range: 3.3–5.5 mmol/L). An electrocardiogram revealed characteristic alterations associated with hyperkalemia, including elevated T waves and an extended QRS duration. The combination of hyponatremia and hyperkalemia, along with clinical symptoms, pointed to inadequate aldosterone production. Additionally, a high serum level of 17-hydroxyprogesterone was detected at 462 ng/mL (normal range: 0.2–0.8 ng/mL).

Considering the laboratory findings and clinical symptoms indicative of the salt-wasting type of CAH, treatment was initiated with intravenous hydrocortisone. The patient was subsequently switched to oral administration of a combination of hydrocortisone (10 mg/day) and fludrocortisone (0.1 mg/day). On the 14th day of hospitalization, the infant was discharged from the hospital in good clinical condition with normalization of laboratory values. He then continued his follow-up care in the pediatric endocrinology unit.

Throughout the follow-up period, there were multiple episodes of acute adrenal insufficiency triggered by acute respiratory viral infections and gastroenteritis. The onset of adrenal crises was alleviated by administering hydrocortisone injections.

During the most recent long-term assessment, a clinical examination, growth monitoring, and laboratory tests were conducted, all of which returned normal results. 17-hydroxyprogesterone, laboratory, and anthropometric measurements during the 4-year follow-up period are detailed in Table 1.

### 2.2. Methodology

#### 2.2.1. Amplicon and Sanger Sequencing

Genetic analysis of *CYP21A2* was performed by amplicon sequencing. Genomic DNA was isolated from whole blood samples. Long-range PCR products were generated with *CYP21A2* gene-specific primers, according to the standard protocol previously described [9]. Amplicons were subjected to sequencing using the Illumina MiSeq platform with the v3 reagents kit, generating paired-end reads of 2 × 300 bp. The average amplicon sequencing depth was more than 500×. Reads were trimmed with fastp (0.23.1) and mapped to the amplicon sequence (chr6: 32037620-32043828 of human reference genome UCSC GRCh38/hg38) by Burrows–Wheeler Aligner software (BWA-MEM2, version 2.2.1) [10,11]. DeepVariant (version 1.6.1) and GATK HaplotypeCaller (4.5.0) were used to call the variants [12,13]. HaplotypeCaller was prioritized for variant analysis, and any discrepancies in pathogenic variants between HaplotypeCaller and DeepVariant were addressed manually. Phasing was carried out using WhatsHap (version 1.7) [14]. Our in-house data interpretation pipeline was used to select prior candidates. Pathogenicity prediction tools, including SpliceAI, AbSplice, and SPiP, were used to predict the functional impact of a candidate variant [15,16,17].

Variant confirmation was performed with Sanger sequencing of PCR products using the following primers: Forward 5′-GAGGCTGAAGCAGGCC-3′ and Reverse 5′-CCCTCTTCCATGCTCGG-3′.

#### 2.2.2. Whole-Genome Sequencing

Genomic DNA was isolated from whole blood samples using the Tecan Freedom EVO automated station (Tecan, Zurich, Switzerland) with the MagAttract HMW DNA Kit (Qiagen, Hilden, Germany). The concentration and purity of the isolated DNA were determined on an Infinite F Nano+ tablet reader (Tecan, Switzerland), and quality assessment was performed using a NanoDrop 8000 (Thermo Fisher Scientific, Waltham, MA, USA).

Sample preparation of genome-wide libraries was carried out using a set of Illumina DNA Prep reagents according to the manufacturer’s recommendations (Document # 1000000025416 v10, Illumina, San Diego, CA, USA) and a set of IDT-ILMN Nextera DNA UD Indexes, Set A and Set B, to prevent cross-contamination of samples. The library concentration was measured on an Infinite F Nano+ tablet reader. The size of the libraries obtained was determined using an Agilent D1000 reagent kit on an Agilent 4200 TapeStation (Agilent Technologies, Santa Clara, CA, USA). Pooling was performed automatically using the Tecan Freedom EVO robotic station (Tecan, Switzerland). Quality control of the pools was carried out using an Agilent MSD 1000 Screen Type reagent kit on an Agilent 4200 TapeStation (Agilent Technologies, Santa Clara, CA, USA). Genome-wide sequencing was performed on an Illumina NovaSeq 6000 instrument using a 300-cycle S4 reagent kit (Illumina, San Diego, CA, USA) for 2 × 150 bp PE reads. The average whole-genome sequencing depth was more than 30×.

Raw reads were trimmed with fastp (0.23.1) and mapped to the human genome reference (UCSC GRCh38/hg38) by Burrows–Wheeler Aligner software (BWA-MEM2, version 2.2.1) [10,11]. Calling was performed with DeepVariant v1.6.1 [12]. The sequenced fragments were visualized using Integrative Genomics Viewer (IGV) software (v2.18.4) [18]. Variants were filtered based on their frequency, being less than 1% in gnomAD (v4.1.0), as well as their effects on coding regions, including missense, nonsense, coding indels, and splice sites [19]. The clinical significance of variants was assessed following the guidelines for interpreting MPS data [20].

### 2.3. Molecular Findings

**Figure 1:**
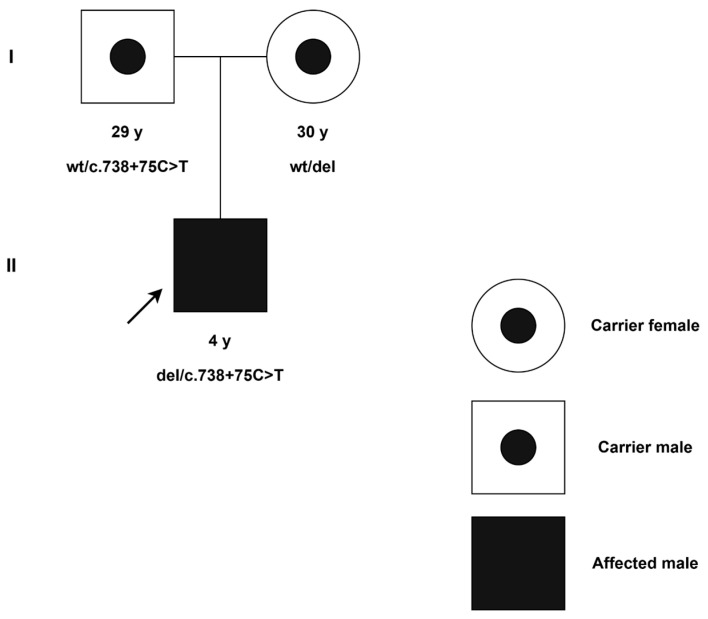
A pedigree for the CAH family. (**I**) generation I; (**II**) generation II. Squares and circles indicate males and females, respectively. The darkened square represents the affected male with hemizygous *CYP21A2*: c.738+75C>T mutation. Black dotted circles represent carriers. The proband is denoted by the slanted arrow.

Amplicon sequencing of the *CYP21A2* gene was carried out for the proband as well as their parents. In the proband, we identified a single copy of the *CYP21A2* gene, with the only potentially deleterious variant on the remaining allele being a hemizygous c.738+75C>T (NM_000500.9) variant located in intron 6. The father was identified as a carrier of the c.738+75C>T (NM_000500.9) mutation in the heterozygous state. For the mother, we were able to conclude that she possessed only one copy of the *CYP21A2* gene due to the absence of heterozygous variants in her amplicon. Such an absence can be caused by either having one gene copy of *CYP21A2* or having two completely similar copies of *CYP21A2*. However, the proband also had no heterozygous variants and inherited only the father’s variants, which would be impossible if the proband inherited any of the mother’s copies. Thus, we concluded that the mother possessed only one copy of *CYP21A2*, and the proband inherited an allele with a missing copy. The proband carried both a paternally inherited intronic mutation and a maternally inherited gene deletion (Figure 1).

The c.738+75C>T (NM_000500.9) mutation is extremely uncommon in population databases, with no instances of homozygotes or hemizygotes found in the gnomAD v4.1.0 and UK Biobank (Allele Frequency Browser) databases [19,21]. However, the frequency data for this variant in population databases is considered unreliable due to the presence of homologous sequences, such as pseudogene *CYP21A1P*, in the genome. The variant has been classified as a variant of uncertain significance (VUS) in the ClinVar database by two laboratories [22].

Pathogenicity prediction tools were used to predict the functional impact of a candidate variant. SpliceAI predicted the variant would cause a Donor Gain (DG) with a moderate pathogenicity likelihood (delta score of 0.65) [15]. AbSplice indicated a moderate probability of pathogenicity in the adrenal gland tissue (score of 0.052), while SPiP suggested a 24% risk of introducing a new splice site [16,17]. The combined results of splicing prediction tools support the hypothesis that the variant contributed to aberrant splicing through the creation of a non-canonical splice site.

According to the ACMG criteria [20] and ClinGen specifications to the ACMG/AMP variant classification guidelines [23], the variant was classified as likely pathogenic (PM3_moderate, PM2_supporting, PP3_supporting, PP4_moderate).

**Figure 2:**
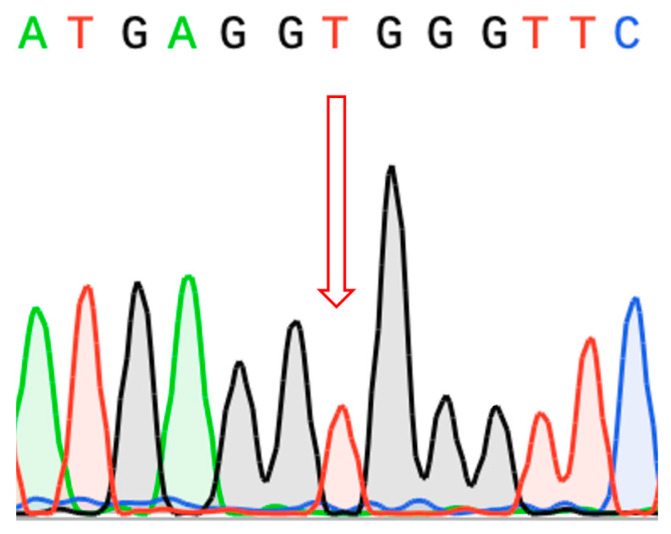
Sanger sequencing of PCR products. Proband carries pathogenic hemizygous variant c.738+75C>T (NM_000500.9).

In addition to amplicon sequencing, whole-genome sequencing (WGS) data were analyzed for all family members to rule out the presence of pathogenic variants in genes that could result in a similar phenotype, particularly *CYP11B1* and *CYP11A1*. Sanger sequencing of the resulting amplicons confirmed hemizygous variation in the proband (Figure 2).

## 3. Discussion

Congenital adrenal hyperplasia (CAH) resulting from 21-hydroxylase deficiency is one of the most prevalent autosomal recessive disorders. However, diagnosing it genetically can be challenging due to the significant variability at the *CYP21A2* locus, which results in a complex array of genetic variations, including rare and novel mutations.

The current best practice approach to genotyping involves Sanger sequencing in combination with MLPA, with Sanger sequencing being performed on the amplicons of the functional *CYP21A2* gene fragments using primers differentiating it from the *CYP21A1P* pseudogene. While MLPA is a sensitive tool for detecting gene conversions and copy number variations, PCR-based Sanger sequencing usually focuses solely on the exons and their adjacent exon/intron boundaries. As of 2020, only a minority of laboratories include sequencing of the 5′ and 3′ untranslated regions (UTRs), while deep intronic regions generally remain unexamined [24].

Compared to Sanger sequencing, next-generation sequencing (NGS)-based methods allow for wider coverage of the genome depending on the specific design of the experiment. In *CYP21A2* sequencing, the most challenging part remains separation of the functional gene from its *CYP21A1P* pseudogene, which is generally performed via long-range PCR using specific primers from [9]. This approach amplifies both *CYP21A2* and most of the chimeric *CYP21A2*/*CYP21A1P* genes, and allows direct determination of its full sequence including all intronic regions [25].

It is important to note, however, that such an approach in the case of the chimeric gene can lead to the detection of multiple heterozygous pseudogene-derived pathogenic variants that, for the purpose of CAH diagnosis, require phasing. Apart from segregation analysis in parental samples, this problem can be mitigated via additional locus-specific amplification [26] or long-read sequencing [27]. In this work, we used trio-based amplicon sequencing to enable a more accurate determination of the origin of genetic variants and allow for confirmation of carrier status in the parents.

**Figure 3:**
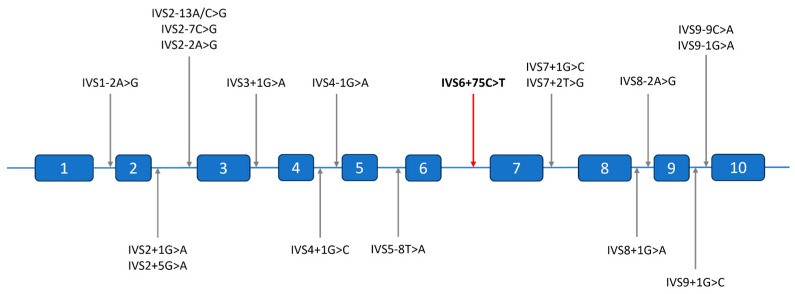
Distribution of intronic pathogenic variants identified in the *CYP21A2* gene according to the literature. The new variant position is indicated by a red arrow.

To date, 18 intronic variants, accounting for 7.7% of all known disease-causing variants, have been identified in the *CYP21A2* gene [28,29]. In silico analyses or functional studies have demonstrated that all these variants are linked to the severe form of 21-hydroxylase deficiency, as they alter the gene’s reading frame, resulting in a non-functional enzyme [30,31]. Typically, an intronic variant disrupts the acceptor or donor site, leading to the activation of an intronic cryptic acceptor or donor site, retention of an entire intron or part of it, and exon skipping. Five variants have been identified within intron 2, making it the most affected intron of the gene [30]. Among them, the IVS2-13A/C>G mutation, originating from the *CYP21A1P* pseudogene, is the most common allele responsible for the SW form of 21-hydroxylase deficiency [32]. All variants described in the literature or genomic variation databases are located in the canonical dinucleotide splice sites (donor GT and acceptor AG) or standard splice regions (−3/+6 for donor and −20/+1 for acceptor site motif), and there have been no reports of pathogenic variants in the deeper intronic regions to date (Figure 3).

In this study, WGS, amplicon, and Sanger sequencing were used for the genetic evaluation of a proband and family members with no family history of CAH. We clarified the pathogenic nature of the variant c.738+75C>T (rs1463196531) and identified its association with the severe phenotype. The variant is situated 75 nucleotides from the closest exon-intron junction and represents the first confirmed pathogenic variant within intron 6 of the *CYP21A2* gene. With the IVS2-13A/C>G variant being the most distant from the nearest exon–intron junction described in the literature, the novel variant is beyond the usual splice site intervals and would generally fall outside exon-based genotyping strategies, including Sanger sequencing.

**Figure 4:**
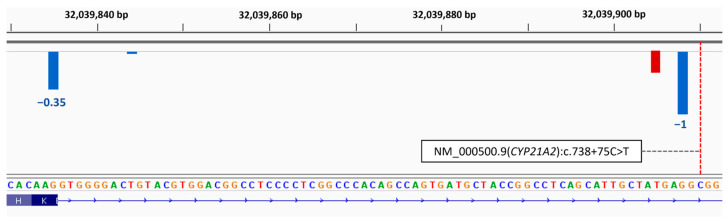
SpliceAI visual outputs of the *CYP21A2* intronic variant displayed in IGV [33]. SpliceAI raw scores for the reference allele and pathogenic variant NM_000500.9 (*CYP21A2*): c.738+75C>T, which is responsible for the inclusion of an intronic sequence containing a stop codon (red). Blue: donor site prediction. The variant position is indicated by a dashed red line.

Multiple pathogenicity prediction tools suggested that this variant causes abnormal splicing, as the new donor splice site score is higher than the canonical one (SpliceAI—delta score of 0.65, SPiP—24% risk, and AbSplice—score of 0.052; for detailed information see Section 2.3. It is anticipated that this variant is responsible for the inclusion of an intronic sequence containing a stop codon at position c.738+70* (TGA), resulting in a transcript’s degradation by nonsense-mediated mRNA decay (Figure 4). As such, this variant is expected to be associated with the complete loss of function of the corresponding protein and salt-wasting CAH in a homozygous state. However, functional studies have not yet been conducted to confirm this effect.

Although previous studies evaluated the splicing effects of various *CYP21A2* variants using cellular models, such as minigene assays and the transfection of mutant constructs, these specialized functional analyses were outside the scope of the present report [30,31].

It is commonly thought that the phenotype is shaped by the allele that has a milder impact. Patients with salt-wasting CAH usually carry complete loss-of-function mutations on both alleles. As our subject has a complete loss of one copy of the gene, the remaining intronic allele, being in a hemizygous state, is likely to result in a total loss of function as well.

To enhance public health and reduce the burden on families and society, it is essential to incorporate genetic compatibility testing into pregnancy planning for prospective parents. Analyzing the *CYP21A2* gene sequence plays a vital role in carrier screening and disease diagnosis, which is essential for preventing adrenal crises and reducing mortality in newborns with the salt-wasting form of CAH.

## 4. Conclusions

In conclusion, our case may provide insights into the phenotype associated with the c.738+75C>T mutation in the *CYP21A2* gene, which is suggested to be linked to the salt-wasting form of congenital adrenal hyperplasia. As this variant lies outside typical regions covered by Sanger sequencing, we recommend comprehensive sequencing of the entire *CYP21A2* gene for patients exhibiting clinical and hormonal signs of 21-hydroxylase deficiency, particularly when there is a mismatch between Sanger- or MLPA-obtained genotypes and phenotypes. Given that CAH clinical manifestations can result from diverse variant types, thorough genetic analysis is essential. In this context, family trio-based long-range PCR amplicon sequencing is a valuable tool to detect mutations that fall beyond the reach of standard diagnostic techniques for genetic analysis of 21-hydroxylase deficiency.

## Figures and Tables

**Table 1:** The evolution of anthropometric and laboratory measurements of the patient.

Laboratory Values	Normal Range	2020 June (Diagnosis)	2021 July	2022 June	2022 October	2023 April	2023 August	2023 September	2024 September (Last Follow-Up)
Ongoing treatment at the moment of presentation (mg/day)	–	–	HC—5 FLC—0.1	HC—5 FLC—0.83	HC—3.75 FLC—0.066	HC—3.75 FLC—0.066	HC—3.75 FLC—0.066	HC—7.5 FLC—0.066	HC—7.5 FLC—0.066
Na^+^ (mmol/L)	132–147	120	138	–	138	–	–	138	139
K^+^ (mmol/L)	3.6–6.1	9	4.4	–	3.9	–	–	3.8	4.5
17-hydroxyprogesterone (ng/mL)	0.2–0.8	462	0.08	0.1	0.53	4.74	41.5	2.36	0.15
Glucose (mmol/L)	3.3–5.5	2.5	5	–	4.35	–	–	3.7	5
Height (cm)	age dependent	50	73 (−1.26 SDS)	–	89 (−0.24 SDS)	–	–	98 (−0.1 SDS)	107 (+0.36 SDS)
Weight (kg)	age dependent	3310	8.9	–	13.6	–	–	13.6	19.3
BMI (kg/m^2^)	age dependent	12.7	16.5	–	17.2	–	–	14.2	16.9

Abbreviations: HC, hydrocortisone; FLC, fludrocortisone.

## Data Availability

Original contributions presented in this study are included in the article/. Further inquiries can be directed to the corresponding author(s).

## Abbreviations

The following abbreviations are used in this manuscript:

CAH	Congenital adrenal hyperplasia
HC	Hydrocortisone
FLC	Fludrocortisone
NGS	Next-generation sequencing
WGS	Whole-genome sequencing
IGV	Integrative Genomics Viewer
MPS	Massive parallel sequencing
MLPA	Multiplex ligation-dependent probe amplification

## References

[1] Parsa A.A., New M.I. (2017). Steroid 21-Hydroxylase Deficiency in Congenital Adrenal Hyperplasia. Genet. Steroid Disord..

[2] Merke D.P., Bornstein S.R. (2005). Congenital Adrenal Hyperplasia. Lancet.

[3] Riedl S., Röhl F.-W., Bonfig W., Brämswig J., Richter-Unruh A., Fricke-Otto S., Bettendorf M., Riepe F., Kriegshäuser G., Schönau E. (2019). Genotype/Phenotype Correlations in 538 Congenital Adrenal Hyperplasia Patients from Germany and Austria: Discordances in Milder Genotypes and in Screened Versus Prescreening Patients. Endocr. Connect..

[4] Mokrysheva N.G., Melnichenko G.A., Adamyan L.V., Troshina E.A., Molashenko N.V., Sazonova A.I., Uvarova E.V., Esayan R.M., Andreeva E.N., Uzhegova Z.A. (2021). Russian clinical practice guidelines «congenital adrenal hyperplasia». Obes. Metab..

[5] Oelkers W.K. (1996). Effects of Estrogens and Progestogens on the Renin-Aldosterone System and Blood Pressure. XVII Meet. Int. Study Group Steroid Horm..

[6] Kim J.H., Kim G.-H., Yoo H.-W., Choi J.-H. (2023). Molecular Basis and Genetic Testing Strategies for Diagnosing 21-Hydroxylase Deficiency, Including CAH-X Syndrome. Ann. Pediatr. Endocrinol. Metab..

[7] Blanchong C.A., Zhou B., Rupert K.L., Chung E.K., Jones K.N., Sotos J.F., Zipf W.B., Rennebohm R.M., Yu C.Y. (2000). Deficiencies of Human Complement Component C4a and C4b and Heterozygosity in Length Variants of RP-C4-CYP21-TNX (RCCX) Modules in Caucasians: The Load of Rccx Genetic Diversity on Major Histocompatibility Complex–Associated Disease. J. Exp. Med..

[8] Balsamo A., Baldazzi L., Menabò S., Cicognani A. (2010). Impact of Molecular Genetics on Congenital Adrenal Hyperplasia Management. Sex. Dev..

[9] Lee H.-H., Poliseno L. (2014). Mutational Analysis of CYP21A2 Gene and CYP21A1P Pseudogene: Long-Range PCR on Genomic DNA. Pseudogenes: Functions and Protocols.

[10] Chen S. (2023). Ultrafast One-Pass FASTQ Data Preprocessing, Quality Control, and Deduplication Using Fastp. iMeta.

[11] Vasimuddin M., Misra S., Li H., Aluru S. Efficient Architecture-Aware Acceleration of BWA-MEM for Multicore Systems. Proceedings of the 2019 IEEE International Parallel and Distributed Processing Symposium (IPDPS), Rio de Janeiro.

[12] Poplin R., Chang P.-C., Alexander D., Schwartz S., Colthurst T., Ku A., Newburger D., Dijamco J., Nguyen N., Afshar P.T. (2018). A Universal SNP and Small-Indel Variant Caller Using Deep Neural Networks. Nat. Biotechnol..

[13] Genomics in the Cloud. https://www.oreilly.com/library/view/genomics-in-the/9781491975183/.

[14] Martin M., Patterson M., Garg S., O Fischer S., Pisanti N., Klau G.W., Schöenhuth A., Marschall T. (2016). WhatsHap: Fast and Accurate Read-Based Phasing. bioRxiv.

[15] Jaganathan K., Panagiotopoulou S.K., McRae J.F., Darbandi S.F., Knowles D., Li Y.I., Kosmicki J.A., Arbelaez J., Cui W., Schwartz G.B. (2019). Predicting Splicing from Primary Sequence with Deep Learning. Cell.

[16] Wagner N., Çelik M.H., Hölzlwimmer F.R., Mertes C., Prokisch H., Yépez V.A., Gagneur J. (2023). Aberrant Splicing Prediction across Human Tissues. Nat. Genet..

[17] Leman R., Parfait B., Vidaud D., Girodon E., Pacot L., Le Gac G., Ka C., Ferec C., Fichou Y., Quesnelle C. (2022). SPiP: Splicing Prediction Pipeline, a Machine Learning Tool for Massive Detection of Exonic and Intronic Variant Effects on mRNA Splicing. Hum. Mutat..

[18] Robinson J.T., Thorvaldsdóttir H., Wenger A.M., Zehir A., Mesirov J.P. (2017). Variant Review with the Integrative Genomics Viewer. Cancer Res..

[19] Chen S., Francioli L.C., Goodrich J.K., Collins R.L., Kanai M., Wang Q., Alföldi J., Watts N.A., Vittal C., Gauthier L.D. (2024). A Genomic Mutational Constraint Map Using Variation in 76,156 Human Genomes. Nature.

[20] Richards S., Aziz N., Bale S., Bick D., Das S., Gastier-Foster J., Grody W.W., Hegde M., Lyon E., Spector E. (2015). Standards and Guidelines for the Interpretation of Sequence Variants: A Joint Consensus Recommendation of the American College of Medical Genetics and Genomics and the Association for Molecular Pathology. Genet. Med..

[21] Li S., Carss K.J., Halldorsson B.V., Cortes A., UK Biobank Whole-Genome Sequencing Consortium (2023). Whole-Genome Sequencing of Half-a-Million UK Biobank Participants. medRxiv.

[22] VCV000800630.4-ClinVar-NCBI. https://www.ncbi.nlm.nih.gov/clinvar/variation/800630/.

[23] Ellingford J.M., Ahn J.W., Bagnall R.D., Baralle D., Barton S., Campbell C., Downes K., Ellard S., Duff-Farrier C., FitzPatrick D.R. (2022). Recommendations for Clinical Interpretation of Variants Found in Non-Coding Regions of the Genome. Genome Med..

[24] Baumgartner-Parzer S., Witsch-Baumgartner M., Hoeppner W. (2020). EMQN Best Practice Guidelines for Molecular Genetic Testing and Reporting of 21-Hydroxylase Deficiency. Eur. J. Hum. Genet..

[25] Shiryagin V.V., Devyatkin A.A., Fateev O.D., Petriaikina E.S., Bogdanov V.P., Antysheva Z.G., Volchkov P.Y., Yudin S.M., Woroncow M., Skvortsova V.I. (2024). Genomic Complexity and Clinical Significance of the RCCX Locus. PeerJ.

[26] Gangodkar P., Khadilkar V., Raghupathy P., Kumar R., Dayal A.A., Dayal D., Ayyavoo A., Godbole T., Jahagirdar R., Bhat K. (2020). Clinical Application of a Novel next Generation Sequencing Assay for CYP21A2 Gene in 310 Cases of 21- Hydroxylase Congenital Adrenal Hyperplasia from India. Endocrine.

[27] Li H., Zhu X., Yang Y., Wang W., Mao A., Li J., Bao S., Li J. (2023). Long-read sequencing: An Effective Method for Genetic Analysis of CYP21A2 Variation in Congenital Adrenal Hyperplasia. Clin. Chim. Acta.

[28] Speiser P.W., Azziz R., Baskin L.S., Ghizzoni L., Hensle T.W., Merke D.P., Meyer-Bahlburg H.F.L., Miller W.L., Montori V.M., Oberfield S.E. (2010). Congenital Adrenal Hyperplasia Due to Steroid 21-Hydroxylase Deficiency: An Endocrine Society Clinical Practice Guideline. J. Clin. Endocrinol. Metab..

[29] Concolino P., Rizza R., Costella A., Carrozza C., Zuppi C., Capoluongo E. (2017). CYP21A2 Intronic Variants Causing 21-Hydroxylase Deficiency. Metab.-Clin. Exp..

[30] Katsumata N., Shinagawa T., Horikawa R., Fujikura K. (2010). Novel Intronic CYP21A2 Mutation in a Japanese Patient with Classic salt-Wasting Steroid 21-Hydroxylase Deficiency. Metab.-Clin. Exp..

[31] Rubtsov P.M., Igudin E.L., Pichugina M.Y., Spirin P.V., Prassolov V.S., Tyul’pakov A.N. (2011). Characterization of a New Splicing Mutation in the Steroid 21-Hydroxylase Gene. Russ. J. Bioorg. Chem..

[32] Kocova M., Concolino P., Falhammar H. (2022). Characteristics of In2G Variant in Congenital Adrenal Hyperplasia Due to 21-Hydroxylase Deficiency. Front. Endocrinol..

[33] Agathe J.-M.d.S., Filser M., Isidor B., Besnard T., Gueguen P., Perrin A., Van Goethem C., Verebi C., Masingue M., Rendu J. (2023). SpliceAI-visual: A Free Online Tool to Improve SpliceAI Splicing Variant Interpretation. Hum. Genom..

